# Is any wheelchair better than no wheelchair? A Zimbabwean perspective

**DOI:** 10.4102/ajod.v4i1.201

**Published:** 2015-11-20

**Authors:** Surona Visagie, Tecla Mlambo, Judith van der Veen, Clement Nhunzvi, Deborah Tigere, Elsje Scheffler

**Affiliations:** 1 Centre for Rehabilitation Studies, Stellenbosch University, South Africa; 2 College of Health Sciences, University of Zimbabwe, Zimbabwe; 3 CBM Africa, East London, South Africa

## Abstract

**Background:**

Within a rights-based paradigm, wheelchairs are essential in the promotion of user autonomy, dignity, freedom, inclusion and participation.

**Objectives:**

This paper aimed to describe a group of Zimbabwean wheelchair users’ satisfaction with wheelchairs, wheelchair services and wheelchair function.

**Method:**

A mixed method, descriptive study was done. Quantitative data was collected from 94 consecutively sampled wheelchair users, who accessed wheelchair services at 16 clinics in five Zimbabwean provinces between October 2013 and February 2014, using the Quebec User Evaluation of Satisfaction with Assistive Technology for adults and children and Functioning Every day with a Wheelchair questionnaire. Qualitative data were collected through two focus group discussions (22 participants) and two case studies with participants purposively sampled from those who participated in the quantitative phase.

**Results:**

More than 60% of participants were dissatisfied with the following wheelchair features: durability (78.6%), weight (75.6%), ease of adjustment (69.1%), effectiveness (69.0%), safety (66.7%), reliability (66.7%), and meeting user needs (60.6%). Similarly, more than 66% of participants were dissatisfied with various services aspects: professional services (69.0%), follow-up (67.0%), and service delivery (68.3%). Although 60% of participants agreed that the wheelchair contributed to specific functions, more than 50% of participants indicated that the features of the wheelchair did not allow in- (53.2%) and outdoor (52.7%) mobility.

**Conclusion:**

Findings indicate high levels of dissatisfaction with wheelchair features and services, as well as mobility. It is recommended that policy and minimum service standards which incorporate evidence and good practice guidelines for wheelchair services and management of wheelchair donations are developed for Zimbabwe.

## Introduction

According to Borg, Larsson and Ö stergren (2011:165), assistive technology, which includes wheelchairs, ‘has been a missing bridge along the road to human rights and development … for many people, particularly in low-income countries’. The United Nations Convention on the Rights of Persons with Disabilities (UNCRPD) (UN 2006) specifically refers to the provision of assistive technology in five articles (Borg *et al*. 2011), with Article 20 focusing on personal mobility (UN 2006). For people with no or limited ability to walk, wheelchairs can enhance function and independence and may open opportunities for work and leisure which otherwise might have been impossible. Within a rights-based paradigm, wheelchairs are therefore essential tools in the promotion of user autonomy, dignity, freedom, inclusion and participation (Borg *et al*. 2011).

Function in a wheelchair is, however, dependent on a complex interaction between user characteristics, activities and social roles, the environment, wheelchair features as well as user assessment and training (Routhier *et al*. 2003). Thus wheelchairs should be appropriate to the users’ functional, environmental, posture support and durability needs (Pearlman *et al*. 2008; WHO 2008). Comprehensive rehabilitation and wheelchair services, as well as trained personnel are instrumental in providing appropriate wheelchairs and achieving right-based outcomes (WHO 2008).

**FIGURE 1:**
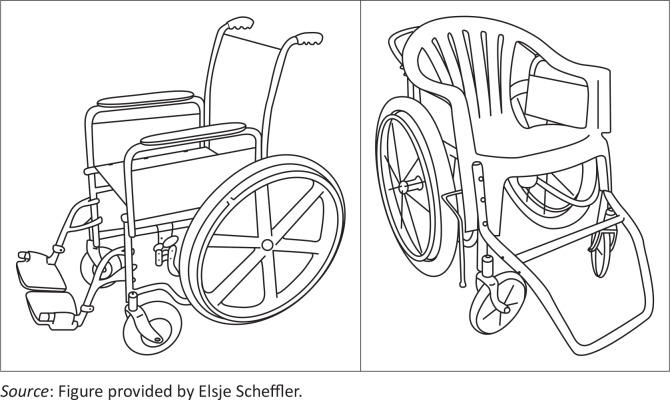
Examples of the basic folding (left) and non-folding (right) wheelchairs provided by donor organisations.*Source:* Figure provided by Elsje Scheffler.

Persons with mobility impairments in Southern Africa have limited access to appropriate wheelchairs and wheelchair services (Eide & Ø derud 2009; Eide *et al*. 2011; Visagie, Scheffler & Schneider 2013) with demand surpassing supply. Donor organisations often attempt to fill this void by donating wheelchairs in bulk, mostly basic folding and basic non-folding frame designs as shown in the examples in Figure 1.

The basic folding frame design is essentially for temporary or low active indoor use. Apart from height adjustable footplates, it lacks adjustability to optimise fit, posture support and function and is not appropriate for active use in less resourced settings (Eide & Ø derud 2009; Mukherjee & Samanta 2005; Pearlman *et al*. 2008; Toro *et al*. 2012). The non-folding wheelchair has a solid seat system, offers no adjustability and has a similar ergonomic design and thus shortcomings as the basic folding frame wheelchair (Mukherjee & Samanta 2005). Despite the limited gains in function and independence offered by these wheelchairs as described by Shore and Juillerat (2012), the limitations of these designs fuel the longstanding and ongoing debate on whether 'something is better than nothing’ (Pearlman *et al*. 2008; Rotary International 2014; WHO 2006).

In January 2012 the Comprehensive Mobility Support Project (CMSP) was implemented in Zimbabwe by the Jairos Jiri Association in partnership with Christian Blind Mission (CBM) and the Ministry of Health and Child Welfare (MOHCW n.d.). Financial support was provided by USAID. The aim of the project was to improve the quality of life of persons with mobility impairments in Zimbabwe by developing capacity and providing appropriate comprehensive wheelchair services. This paper presents baseline data collected during the CMSP and aims to add to the debate on whether any wheelchair is better than no wheelchair by describing satisfaction with wheelchairs, wheelchair services and wheelchair function of a group of Zimbabwean wheelchair users of primarily basic folding and rigid frame wheelchairs.

## Zimbabwean context

**FIGURE 2:**
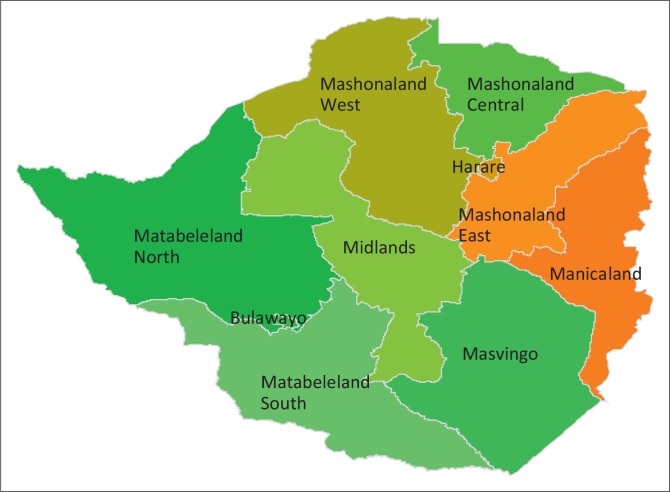
A map of Zimbabwe and its provinces.

Zimbabwe is a landlocked country in Southern Africa covering 390 757 km^2^. It is divided into 10 provinces of which two, Bulawayo and Harare, are cities with provincial status (Figure 2). Zimbabwe has a population of 13 million with a population density of 33 persons per square kilometre. Sixty-seven percent of the population live in rural areas. The life expectancy at birth is 38 years. Approximately 6% of the population live with a disability, of these 35.9% have difficulty moving and might require a wheelchair (ZIMSTAT 2013).

The majority of Zimbabweans are dependent on public healthcare provided by the MOHCW, complemented by non-governmental organisations and a small private sector (MOHCW n.d.). Rehabilitation services are provided by occupational and physiotherapists in urban centres, as well as rehabilitation technicians who are the primary providers in rural areas at the district hospitals and community-based rehabilitation (CBR) programmes. Rehabilitation technicians outnumber therapists at a ratio of approximately 2:1 (Personal communication with Medical Rehabilitation Practitioners’ Council of Zimbabwe, 09/04/2015). A situational analysis by the CMSP team found that Zimbabwe had no policy on wheelchair service provision and that wheelchair services were fragmented and poorly integrated in rehabilitation services. Limited numbers of wheelchairs were provided by hospitals as part of rehabilitation services, or purchased directly from retail shops. However, many wheelchairs were donated by non-governmental organisations, churches and politicians without any clinical or follow-up support services.

## Methods

The study comprised a descriptive design with a quantitative and qualitative phase and is part of a larger mixed method descriptive study with a pre-test post-test component. A sequential explanatory strategy where qualitative data were collected after quantitative data in order to explore and contextualise quantitative findings through the experiences and perceptions of individual participants (Kroll, Neri & Miller 2005) was used. The larger study commenced in October 2013 and was completed in May 2014. This paper focuses on wheelchair users’ experiences prior to the implementation of the Comprehensive Mobility Support Project (CMSP) in 16 clinics. Data presented in this paper were collected between 30 October 2013 and 28 February 2014 at the 16 clinics in the Zimbabwean provinces of Harare, Mashonaland East, Matabeleland South, Bulawayo and Masvingo where the CMSP was to be implemented.

### Quantitative phase

All 135 persons who accessed the selected clinics for a wheelchair between 30 October 2013 and 28 February 2014 were consecutively recruited to participate in the study. In order to be included in the study users had to be basic^1^ or intermediate^2^ level manual wheelchair users and they were to have accessed services at one of the CMSP seating clinics during the study period. Only the findings of the 94 existing wheelchair users are presented in this paper, since they were the only participants with previous experience of wheelchair use and wheelchair service delivery. Data collection tools included a self-designed demographic questionnaire and three standardised questionnaires: the Quebec User Evaluation of Satisfaction with Assistive Technology (QUEST 2.0) for adults (Demers, Weiss-Lambrou & Ska 2000), the QUEST 2.1 for children (Murchland, Kernot & Parkyn 2011) and Functioning Every day with a Wheelchair (FEW) questionnaire (Mills, Holm & Schmeler 2007). The self-designed demographic questionnaire was used to gather socio-demographic and clinical information.

The QUEST 2.0 assesses user satisfaction with assistive technology. Of the 12 questions, eight address satisfaction with the device (dimensions, weight, adjustments, safety, durability, simplicity of use, comfort, and effectiveness) and four address satisfaction with the service provision process (service delivery, repairs/servicing, professional service and follow-up services). The QUEST 2.0 was found valid and reliable in Global North settings (Demers *et al*. 2002). The children`s version (QUEST 2.1) was derived from the QUEST 2.0 and validated for use with children in a Global North setting (Murchland *et al*. 2011). Three features, namely ease of adjustment, safety and comfort, are not included in the QUEST 2.1 for children. Instead, it includes ease to move, appearance and time required to set up the device. Adult users rate their satisfaction with various features of the device on a 5-point Likert scale. According to the QUEST 2.0, manual, items which require attention are those where ’at least 25% to 33% of users report that they are only “somewhat satisfied”, ”dissatisfied”, or ”very dissatisfied”’ (Demers *et al*. 2000:28). The 5-point rating scale of the QUEST 2.0 was therefore collapsed into two categories, that is, ‘quite or very satisfied’, and ‘somewhat satisfied’, ‘dissatisfied’, or ‘very dissatisfied’ as was done by previous authors (Bergstrom & Samuelsson 2006; Samuelsson & Wressle 2008). Children use a 7-point pictorial Likert scale. Users also select the three features they consider most important.

The FEW assesses users’ perceptions of the impact of the wheelchair on their function. Ten items are rated on a 6-point Likert scale with an additional ‘Does not apply’ option. The FEW has been found to capture 96.9% to 99.7% of users’ goals in wheelchair use with moderate precision for test-retest reliability (Mills **et al*.* 2007).

The data collection tools were translated into Shona and Ndebele, the main local languages in Zimbabwe. The forward translations were done by two qualified occupational therapists who were native Shona and Ndebele speakers. A multi-linguist from the Medical Research Council of Zimbabwe reviewed and compared both translations to the original English versions for correctness and consistency. Twenty-five trained research assistants collected the data. Of these, 17 were also service providers at the clinics. Quantitative data were coded and entered into Microsoft Excel. Data are presented in percentages and summarised in figures and graphs.

### Qualitative phase

Qualitative data on user satisfaction and function were collected through two focus group discussions and two case studies. The participants were purposively selected by the research team based on perceptions formed about the richness of information they could offer. Discussion guides were used in both focus groups and case study interviews to ensure in-depth exploration of the issues under study. The main topics explored were:

- Participants’ experiences and problems as wheelchair users in life situations
- Satisfaction with their wheelchairs
- How the experience of wheelchair users in Zimbabwe can be improved.

Focus group participants included users, family members/caregivers, and service providers. One focus group discussion was held in a rural setting in Masvingo province in January 2014 with ten participants and the other in an urban setting in Harare province in April 2014 with 12 participants. Each focus group lasted about four hours. The two case study participants included a nine-year-old boy and a 26-year-old woman. Data collection included participant observation and in-depth interviews over a number of visits during the study period.

The focus group discussions and case study interviews were audio-recorded and transcribed verbatim. As the purpose of the qualitative data was to explore and contextualise quantitative findings narrative examples from the transcripts are presented with the quantitative results.

#### Ethical considerations

Ethical approval was granted by the Joint Research Ethics Committee (JREC/323/13) of the University of Zimbabwe, College of Health Sciences and by the Medical Research Council of Zimbabwe (MRCZ/A/1813). Written informed consent from wheelchair users, parents, guardians or caregivers, as appropriate, as well as assent from child participants was sought. Parents, guardians and/or caregivers became proxy respondents for adult and child participants who were unable to communicate or understand due to the nature of their disabilities. The informed consent documents included permission to audio record focus group and case study interviews. Participation was voluntary and participant privacy and confidentiality were maintained.

## Results

### Demographic information

Fifty (53%) of the participants were children and 44 (47%) adults. The median age of the study participants was 16 years (interquartile range: 11 to 42). The majority (57%) were men. Forty-nine percent of the participants had cerebral palsy (Table 1). Sixteen percent of adult participants were either formally or informally employed. Of the 50 children, 29 (58%) were attending school. Reasons for not attending school included no suitable school or resources to accommodate learners using wheelchairs, a lack of transport, the nature of the disability, parents not seeing the need for schooling, and financial challenges. The majority of users 47 (50%) were living in rural areas with 39 (42%) living in urban areas and the rest living in peri-urban areas.

**TABLE 1:** Health conditions necessitating the use of a wheelchair (*n* = 94).

Condition	% Total	% of children (*n* = 50)	% of adults (*n* = 44)
Cerebral palsy	49	74	23
Paraplegia/Spinal cord injury (SCI)	16	4	30
Polio	10	2	18
Stroke	2	0	4
Muscular dystrophy	5	6	2
Amputation	3	0	7
Other	11	14	7
Unknown	4	0	9

The majority (79%) of participants was dependent on public transport, whilst 9% used private transport and 12% used both modalities. The qualitative data particularly highlighted how non-folding wheelchair designs resulted in users being excluded from community participation through transport challenges.

… if you cannot fold it … they *(transport crew)* won`t allow you in … they don`t have space for it … you are then forced not to travel … (Woman, 26, user)

… with the non-foldable, my major challenge is with transportation … when the chair can`t fit in the commuter-omnibus, I am forced to leave it when travelling … travelling without the wheelchair … you can just imagine! (Man, 27, user)

Folding designs were more readily transported:

… and the fact that it's foldable is very important for us … it means we can put it in a ’combi’ (commuter omnibus) and we can go to church with her … (Woman, 39, caregiver)

### Wheelchair provision

Wheelchairs were primarily (45%) supplied by rehabilitation technicians, followed by wheelchair technicians (5%) and therapists (5%) (Table 2). Others included two doctors, a headmistress, a carpenter and relatives/family/friends. Twenty-seven percent of participants did not know who supplied their wheelchair. The majority of participants used a wheelchair with a basic folding and non-folding design (Figure 1). As the impact of these wheelchairs on user satisfaction and function was studied, the global effect of these common wheelchair features are presented, rather than information related to numbers and types of wheelchairs.

**TABLE 2:** Profession of person supplying the wheelchair as reported by users (*n* = 94).

Provider	% Total
Physiotherapist	3
Occupational therapist	2
Rehabilitation technician	45
Orthopaedic technologist	1
Wheelchair technician	5
Other	17
Did not know	27

The majority of the study participants (89.7%) had received their wheelchair as a donation, 8% had bought it from a retailer, and 2.3% had borrowed a wheelchair. Although challenges of fit, posture support, function and safety were often mentioned, users expressed gratitude and satisfaction at being offered mobility. 9-year-old Jay's (case study participant) mother explained her satisfaction with a donated wheelchair despite needing someone almost full time to frequently reposition Jay and to assist him to move around: ‘Satisfaction with the wheelchair was high, maybe because we didn’t know what to expect from it other than to ferry Jay around’ (Woman, 47, caregiver).

According to focus group participants, wheelchair provision was not supported by a comprehensive wheelchair service. They received no formal assessment, prescription, fitting or training, and both maintenance and follow-up services were limited.

… to function well … you need proper training in real life settings, even advice given at the clinic is not enough … with my donated wheelchair, I didn`t get any training and it was not easy to use it … (Man, 35, User)

### Satisfaction of adult users

**FIGURE 3:**
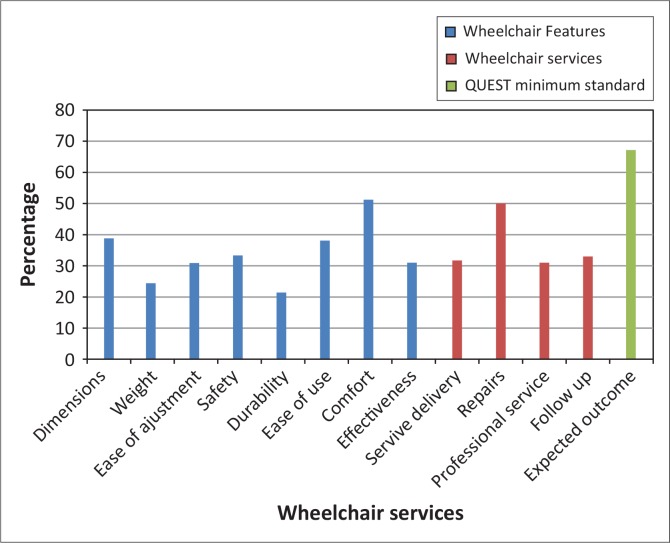
Percentage of adult users who were very or quite satisfied with their wheelchair and wheelchair services according to the QUEST 2.0 (*n* = 44).

More than 60% of adults were dissatisfied with every feature of their wheelchair, except for comfort, which 51% found satisfactory. Dissatisfaction was especially high with durability (78.6%), weight (75.6%), ease of adjustment (69.1%), effectiveness (69%) and safety (66.7%) (Figure 3).

Qualitative data highlighted the effect of poor durability on function: ‘Inflatable tyres lose pressure easily and you don`t get where you want to go or do what you want to do …’ (Man, 41, user).

Poor durability, combined with limited repair services and knowledge in maintenance may have safety implications: ‘My wheelchair broke down some time back and I was tying it with rags’ (Woman, 63, user).

Users associated wheelchair design with durability, albeit limited to rigidity only: ‘… foldable ones are not durable, but they work best when it comes to transportation and access …’ (Man, 44, user and provider).

The fixed foot- and armrests on some wheelchairs limited certain functions, whilst the limited size ranges affected fit, comfort and posture support: ‘My wheelchair was inappropriate for me’; ‘It was too small and I could hardly endure sitting in it for long’. (Woman, 39, user); ‘… the right size with all safety features is important to me … I think it's because I used to fall a lot …’ (Man, 25, user).

Although half the participants were satisfied with repair services, more than two-thirds were not satisfied with service delivery, professional services and follow-up (Figure 3). Qualitative data showed that users were not consulted about their needs, nor did they receive appropriate training; ‘We need to be asked about our environments so that we get what works there …’ (Man, 27, user); ‘Mine was sent from outside and I was not taught how it worked’. (Woman, 63, user).

### Satisfaction of child users

**FIGURE 4:**
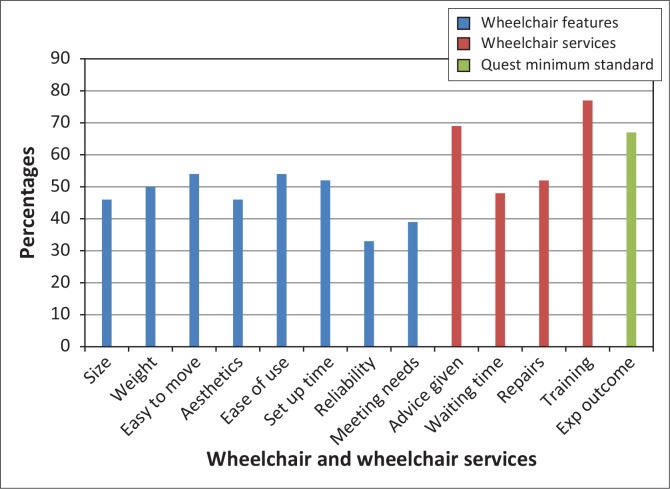
Percentage of child users who were very or quite satisfied with their wheelchair and wheelchair services according to the children's QUEST 2.1 (*n* = 50).

Compared to adults, the child and caregiver QUEST 2.1 scores demonstrate similar dissatisfaction with wheelchair features but higher satisfaction with wheelchair services (Figure 4). However, apart from training, more than 25% of users were not satisfied with services. The two wheelchair features most children and caregivers were satisfied with were the ease of using (53.8%) and moving (53.9%) the wheelchair, whilst 52% were satisfied with how much time it took to set up the wheelchair. Narrative examples underscore this: I am happy that the wheelchair is not difficult to propel but it's too big for my child’ (Woman, caregiver).

However, child users who independently propelled reported that they found their wheelchairs clumsy, heavy and difficult to use. The highest levels of dissatisfaction were reported for reliability (66.7%), meeting user needs (60.6%), appearance (53.9%) and size (53.8%). The wheelchairs were generally too big for the children. Inappropriate wheelchairs and size affected posture support, comfort, function and safety. Most of the children had only basic wheelchairs, with no posture support: ‘I am not happy at all with this wheelchair, it is too big and my child keeps slipping out and falls often’ (Woman, 30, caregiver).

The impact of the environment and wheelchair design on durability was recognised as qualitative data show and, similar to the adults, was associated with rigidity of the frame only: ‘… at times it's not about how strong the chair is … its damaged more from where we use it … the environment is just bad … (Woman, 30, caregiver); ‘… although the non-foldable one has its problems in transportation … I prefer that it's strong …’ (Woman, 37, caregiver).

Although not satisfied with how long it took to receive the wheelchair, child users and their caregivers were generally satisfied with service delivery and professionalism, with 68.8% satisfied with advice and 76.5% satisfied with training provided. Their dissatisfaction with repair services was similar to that of adults (Figures 3 and 4). They qualified their expectations in the qualitative data; ‘… the places for repairs and service should be brought closer to us and should be for free …’ (Woman, 30, caregiver).

Adult users identified durability (55%), comfort (40%) and safety (40%) as priorities for the wheelchair; whereas children/caregivers identified dimensions (56%), ease of use (52%) and meeting their needs (42%) as the most important aspects.

### Function

The extent to which both child and adult participants agreed that the wheelchair facilitates function is presented in Table 3. Scores were collapsed into agree (completely, mostly, slightly agree) and disagree (completely, mostly and slightly disagree). The majority of users (82%) agreed that the wheelchair allowed them to reach and carry out activities at different surface heights. Approximately two-thirds of users felt the wheelchair contributed to daily routines and matched their health needs, and just over 60% agreed that it allowed them to do transfers and personal care tasks. However, fewer than 50% of users indicated that the wheelchair matched their comfort needs, assisted indoor and outdoor mobility or allowed use of transport. Frequent falls were reported for both children and adults. Users prioritised mobility, safety and function: ‘… when I am safe I move faster and I am confident to do it …’ (Man, 44, user and provider).

**TABLE 3:** Functioning every day with a wheelchair (FEW) scores (*n* = 94).

Wheelchair size, fit, posture support & functional features	Agree	Disagree	Does not apply
Contribute to carrying out daily routines	65.6	23.6	10.8
Match comfort needs	49.5	44.1	6.4
Match health needs	66.7	26.9	6.4
Allow safe and efficient independent operation	58.7	29.3	12
Allow reaching and carrying out tasks at different surface heights	82	2.6	15.4
Allow transfers	62.6	21	16.4
Allow carrying out personal care tasks	60.2	23.7	16.1
Allow getting around indoors	46.8	37.2	16
Allow getting around outdoors	47.3	39.9	12.8
Allow use of personal or public transportation	45.2	40.8	14

Ill-matched wheelchair features not only impact on durability, function and safety, but may also impact on the users’ basic human rights, dignity and inclusion, as illustrated by qualitative data:

‘What is important is for my sister to get a chair with tubeless tyres so that we can bring her here (clinic) without having to put her in a wheelbarrow’. (Focus group participant, caregiver)

Some providers reported on the impact of the environment on function, safety and outdoor mobility in the focus groups without considering the impact of the wheelchair design and features, whilst others erroneously associated rigidity, rather than wheelchair design features such as rear wheel adjustability or wheelbase with improved performance and safety.

‘… generally the outdoor environment is not ready for users. You will see … that most can’t even use their wheelchair in their own yard’. (Man, 34, provider)

‘… the environment is just not user friendly and to talk of full functional independence in this context … I just don’t know …’ (Woman, 40, provider)

‘… although the rigid one is not preferred by many, we prescribe it often because safety and posture support are a priority for us …’ (Referring to local rigid frame three-wheeler) (Man, 33, provider)

## Discussion

Although more than 60% of wheelchair users agreed that the wheelchair contributed to specific functions such as reaching and doing tasks at different heights, carrying out daily routines and personal care tasks as well as doing transfers, less than 50% agreed that it allowed them appropriate indoor and outdoor mobility (FEW scores). This finding was echoed in the respective adult and child QUEST items rating on satisfaction with wheelchair performance, namely effectiveness (Figure 3) and meeting user needs (Fig 4), where only 31% of adults and 39.4% of children were satisfied. Most everyday tasks, although performed in various settings, do not require much mobility in the wheelchair, and just being able to sit might assist in performing the tasks. Shore and Juillerat (2012) reported similar improvements in function with a basic non-folding wheelchair for users from Vietnam, India and Chile, whilst Mukherjee and Samanta (2005) reported similar mobility restrictions in a group of Indian wheelchair users of basic non-folding wheelchairs. Considering that a wheelchair is primarily a mobility aid, it should not only promote functional activities in sitting, but also promote in- and outdoor mobility.

In contrast with current study findings on satisfaction on wheelchair mobility, Bergstrom and Samuelsson (2006) found that 98% of Swedish users with SCI were satisfied with their indoor mobility and 80% with outdoor mobility. More than 60% of users in a regional study from South Africa agreed that the wheelchair allowed satisfactory indoor and outdoor mobility (Visagie, Duffield & Unger 2015). Environmental barriers were not explored in any one of the studies. The Swedish setting was presumably more urban and accessible and may have enhanced the performance of and satisfaction with the wheelchair and explain the higher satisfaction scores in the Swedish study. In contrast, the South African study had higher satisfaction scores than the current study, despite having a similar setting.

**FIGURE 5:**
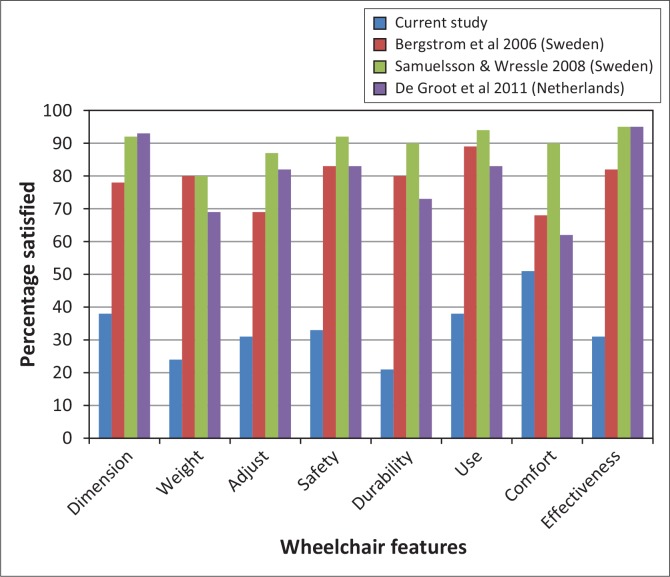
Comparison of QUEST 2.0 wheelchair feature item scores across four studies.

Several features that directly impact on mobility were rated in the QUEST 2.0 and 2.1. Figure 5 shows that, compared to adult users from other studies, users in this study were less satisfied with every wheelchair feature. Although study populations and settings differ, user satisfaction can be compared across these studies, as irrespective of contextual differences, a comprehensive wheelchair service should result in an appropriate wheelchair for each user which meets their functional, posture support, environmental and durability needs.

One explanation for the poor satisfaction with the wheelchair and the challenges with indoor and outdoor mobility found in the current study may be the features of both basic folding and non-folding frame wheelchairs (Figure 1). In these designs the user is positioned relatively high above and in front of the rear wheel axle, compromising efficient propulsion ergonomics, which together with a short wheelbase loads the front castors (Medola *et al*. 2014). The subsequent increase in rolling resistance requires more energy to propel the wheelchair (Medola *et al*. 2014; Mukherjee & Samanta 2005). The loaded castors also get stuck easily against obstacles and in holes or ruts and are difficult to lift when trying to clear these obstacles (Medola *et al*. 2014). Eventually users might lose the ability to push themselves (Ø derud 2014). Wheelchairs with longer wheelbases which reduce the weight on the front castors and ease propulsion on uneven or rough terrain may have increased user satisfaction.

**FIGURE 6:**
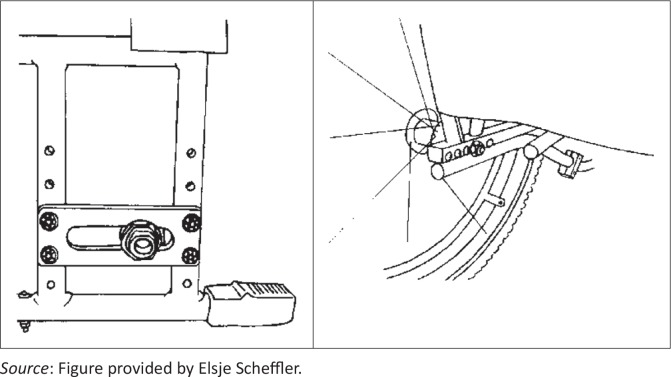
Examples of design options with adjustable rear wheel axle settings. *Source*: Figure provided by Elsje Scheffler.

Furthermore, neither wheelchair offers adjustability to optimise propulsion ergonomics. Wheelchairs with adjustable settings, particularly adjustable rear axle positions (Figure 6), contribute to higher levels of satisfaction and function (Bergstrom & Samuelsson 2006; Karmarkar *et al*. 2009; Medola *et al*. 2014; Rispin & Wee 2015; Samuelsson & Wressle 2008).

**FIGURE 7:**
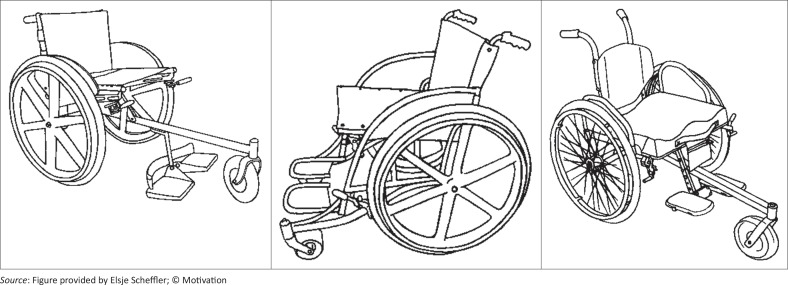
Examples of wheelchair designs referred to in the studies by Visagie *et al*. (2015) and Rispin and Wee (2015). *Source*: Figure provided by Elsje Scheffler; © Motivation

The higher satisfaction levels for outdoor mobility reported by Visagie *et al*. (2015) might be attributable to the features of the particular wheelchair designs used. In their study 43% of users used adjustable wheelchairs with features designed for outdoor environments (Figure 7). Rispin and Wee (2015) demonstrated the superiority of wheelchairs with a long wheelbase and adjustable rear wheel axle settings (Figure 7) in distance travelled, user satisfaction and physiological cost over basic folding frame wheelchairs when tested on rough, uneven tracks. Both users and service providers in the current study ascribed outdoor mobility challenges to only environmental barriers and seemingly failed to recognise the impact that appropriate wheelchair design and features may have on function and mobility. Similarly, they associated improved durability, safety and function of the local three-wheel wheelchairs with rigidity rather than the features such as a long wheelbase and lessened load on the front castors. These findings highlight the need for training of both groups on wheelchair design and how this relates to environment and user needs.

Comfort is an essential wheelchair feature and was ranked as one of the three key features by adults in the current study and other studies (Bergstrom & Samuelsson 2006; Samuelsson & Wressle 2008). Although comfort achieved the highest satisfaction rate (51.2%) in the current study, the percentage of satisfied users was still at least 10 percentage points below that of other studies (Figure 5) (Bergstrom & Samuelsson 2006; De Groot *et al*. 2011; Samuelsson & Wressle 2008). Mukherjee and Samanta (2005) found that comfort was ignored in the distribution of donated wheelchairs in India, resulting in higher dissatisfaction and contributed to wheelchairs being abandoned. Only one previous study reported very high levels of satisfaction with comfort (90%) (Samuelsson & Wressle 2008). In their study 80% of users were satisfied with all wheelchair features reflecting the impact of highly adjustable wheelchairs. The high levels of discomfort reported in the current study may be explained by the wheelchair designs that lack adjustability and an inadequate size range (Ø derud 2014), resulting in poor fit and posture support.

Adult users in the current study ranked durability as the most important feature of the wheelchair. Almost 80% of adults (Figure 3) and 70% of children (Figure 4) experienced durability and reliability problems, which may again be attributable to the use of wheelchairs with basic folding frame (Pearlman *et al*. 2008) and basic non-folding frame designs (Mukherjee & Samanta 2005). This design is not appropriate for active use on uneven terrain such as broken pavement, sand, dirt and mud commonly found in less resourced settings, resulting in undue stress and higher needs for repairs and replacement (Mukherjee & Samanta 2005; Ø derud 2014; Pearlman *et al*. 2008). Disrepair and safety considerations are common reasons for abandoning wheelchairs (Mukherjee & Samanta 2005; Toro *et al*. 2012). Durable wheelchairs have been associated with user satisfaction (Bergstrom & Samuelsson 2006; Visagie *et al*. 2015).

Similar to users in a South African study (Visagie *et al*. 2015), users in the current study were mainly dependent on minibus taxis for transport and experienced challenges related to attitudes, embarking and disembarking, as well as space. Taxi operators often either do not stop for wheelchair users or charge them extra (Cawood 2012; Chakwiriza *et al*. 2010), emphasising the need to consider specific wheelchair features for transport, together with an intersectoral approach to finding solutions for transport challenges.

**FIGURE 8:**
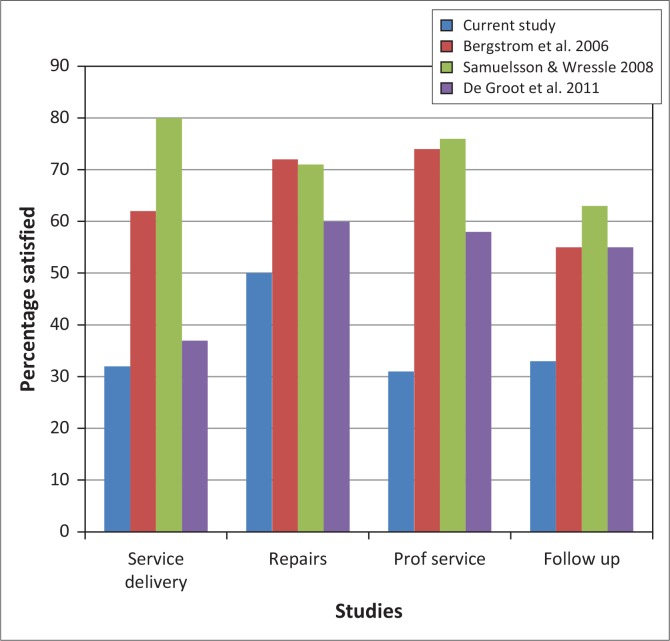
Comparison of QUEST 2.0 wheelchair services item scores across four studies.

The poor satisfaction rates and user comments on service delivery may reflect inadequate training of service providers. Both the UN Convention (UN 2006) and the WHO wheelchair guidelines (WHO 2008) promote comprehensive service delivery by trained service providers. Wheelchair services delivered by well-trained providers have been associated with increased satisfaction amongst wheelchair users (Bergstrom & Samuelsson 2006; Glumac *et al*. 2009; Routhier *et al*. 2003; Samuelsson & Wressle 2008). Users in the current study expressed more dissatisfaction with services compared to other studies (Figure 8). Varying and sometimes conflicting user needs (Bergstrom & Samuelsson 2006; Visagie *et al*. 2015) contribute to the complexity of wheelchair assessment, prescription, fitting and training; thus service personnel require adequate knowledge on wheelchair design, as well as the physical, environmental and psychological needs of the user (Bergstrom & Samuelsson 2006; Glumac *et al*. 2009; Routhier *et al*. 2003; Samuelsson & Wressle 2008; UN 2006; WHO 2008). Although occupational and/or physiotherapists commonly provide wheelchair services in resourced settings (Greer, Brasure & Wilt 2012; Samuelsson & Wressle 2008), rehabilitation technicians were the primary service providers in this study. The WHO wheelchair guidelines (WHO 2008) emphasise suitable training of wheelchair service providers rather than occupation and promote training of other categories of service providers such as community health care workers, community based rehabilitation workers, prosthetists, technicians and craftsmen.

Users were not consulted on their needs, received limited training, and little information. Inadequate service provision in this study negatively impacted on assessment, fit, user training, function and user rights. Visagie *et al*. (2013) demonstrated the negative impact that fragmented services may have on user outcomes. Similarly, Mukherjee and Samanta (2005) and Ø derud (2014) describes how in the absence of comprehensive service delivery, little if any user assessment is done and wheelchairs are provided without consideration of fit, posture support, functional and environmental needs. The subsequent poor match between the wheelchair features and user needs contribute to discomfort, poor mobility, loss of function and stability, poor durability, as well as safety challenges found in the current study and by Mukherjee and Samanta (2005) and Toro *et al* (2012). Adult users in this study ranked safety as one of the top three priorities for their wheelchair. Poor fit and posture support may compromise health needs and can cause secondary complications such as pelvic and trunk deformities and pressure ulcers, a common cause of mortality amongst wheelchair users (Ø derud 2014; Toro *et al*. 2012).

The high mechanical wheelchair failure reported (see durability and reliability items in Figures 3 and 4) necessitates adequate repair services, yet satisfaction with repair services was considerably lower compared to resourced settings (Bergstrom & Samuelsson 2006; De Groot *et al*. 2011; Samuelsson & Wressle 2008) (Figure 8). This may be due to a lack of technical knowledge and spare parts, which are common in less resourced settings (Mukherjee & Samanta 2005; Ø derud 2014; Toro *et al*. 2012). Most accidents in wheelchairs are due to technical malfunction and can be prevented by regular maintenance (Hansen, Tresse & Gunnarsson 2004).

Follow-up services were limited in this study. The 33% satisfaction with follow-up services is again much lower than the rates reported by De Groot *et al*. (2011), Samuelsson and Wressle (2008) and Bergstrom and Samuelsson (2006). Changing user needs, growth or changes in the health conditions make follow-up essential to ensure that problems with fit, posture support, function, durability and safety are identified and addressed (Hansen *et al*. 2004; Samuelsson & Wressle 2008; Toro *et al*. 2012; WHO 2008).

Findings of this study illustrate that donated wheelchairs provided without the necessary comprehensive support services can lead to poor user outcomes. Whilst outside the scope of this study, it is also important to mention that donations provided through a charity model can disempower users rather than promote their rights. Considering the cost of and the need for wheelchairs, many low-resourced settings like Zimbabwe will require donor assistance in its wheelchair service delivery. Donated wheelchairs, if appropriately managed by trained staff and through comprehensive services, can result in satisfactory user outcomes (Glumac *et al*. 2009).

### Limitations

The results of this study must be interpreted with caution due to limitations in the methodology and possible bias. Bias may have been introduced by sampling users who accessed the services, as the extent of the problems they experienced may have been greater than those not accessing the services, culminating into lower levels of satisfaction and function. Drawing a sample from participants who accessed services may have excluded those who could not access services due to contextual barriers.

The standardised tools were not tested for validity and reliability in the study setting or a similar context. Context and culture can influence users’ opinions about what aspects of a device, services or function are important and how people interpret questions and answer options. Thus aspects important to the current study population might have been left unexplored through the tools used.

As some of the data collectors were wheelchair service providers as well, users might have wanted to please data collectors with their answers in order to ensure goodwill for future services or to secure a new wheelchair. Both data collectors and the standard participant information sheet (translated into the two vernacular languages) emphasised that neither refusal nor honest opinions would negatively influence service provision. In all FEW items some users selected the ’does not apply’ option (Table 3). According to data collectors, users chose this option mainly when they were completely dependent in performing the activity or were not full-time wheelchair users. Even so, it is difficult to see how aspects such as comfort and health needs do not apply.

## Recommendations

In light of the challenges identified in Zimbabwe and the discussed positive impact of comprehensive services, trained staff and appropriate wheelchairs on user function and satisfaction, it is recommended that policy and minimum service standards based on evidence and good practice are developed to guide training of wheelchair service providers, wheelchair provision and wheelchair service delivery in Zimbabwe. Wheelchair services in Zimbabwe are heavily dependent on donations. To optimise the outcomes of the impact of these donations it is recommended that these guidelines include management strategies to source and distribute appropriate wheelchair donations through existing service networks. Monitoring and evaluation should form an integral part of the service standards and programme management at service, provincial and national level. The findings also showed a need for access to a wider range of wheelchair design options in order to meet different user functional, posture support and environmental needs. Further studies on the impact of the WHO guidelines (WHO 2008) on wheelchair service delivery, user satisfaction and function are recommended, as is studies on the impact on environmental factors on access to wheelchair services in less resourced settings. A systematic review of studies reporting adult satisfaction with wheelchairs using the QUEST 2.0 is also recommended.

## Conclusion

The study contributes to the body of knowledge on wheelchair user satisfaction and function in less resourced settings. Compared to users from resourced countries, Zimbabwean users were on the whole much less satisfied with their wheelchairs and in particular with their overall mobility, wheelchair durability and comfort as well as wheelchair services. Users were excluded as active participants in the process and, subsequently, were not adequately informed and empowered about wheelchairs and their rights. The study found that, despite high levels of dissatisfaction, inappropriate wheelchairs contributed to some autonomy, freedom and independence in everyday tasks, but simultaneously emphasised the extent to which users` mobility impairment was perpetuated, particularly by failing to meet their environmental, mobility and durability needs. These factors will ultimately limit the users’ inclusion, participation, freedom and independence, whilst simultaneously increasing the risk for injury due to mechanical malfunction. Faced with something versus nothing, or an inappropriate rather than an appropriate wheelchair, users will always be grudgingly grateful: ‘You cannot expect much from a donation, but for you to be thankful’ (Man, 41, User).

But from a rights-based and mobility perspective, wheelchairs, wheelchair services and wheelchair donations should meet the objectives of Article 20 on personal mobility (UN 2006), and place the same value on wheelchairs that users do: ‘The value you put on your legs I place on my wheelchair’ (Woman, 26, user).

## Footnotes

1 Users who can maintain neutral, upright sitting posture without support and need wheelchairs without modification (WHO 2008).

2 Users with mild to moderate postural deviations who need wheelchairs with modifications and supportive seating (WHO 2008).
